# *In vitro* screen of prion disease susceptibility genes using the scrapie cell assay

**DOI:** 10.1093/hmg/ddu233

**Published:** 2014-05-15

**Authors:** Craig A. Brown, Christian Schmidt, Mark Poulter, Holger Hummerich, Peter-C. Klöhn, Parmjit Jat, Simon Mead, John Collinge, Sarah E. Lloyd

**Affiliations:** MRC Prion Unit and Department of Neurodegenerative Disease, UCL Institute of Neurology, Queen Square, London WC1N 3BG, UK

## Abstract

Prion diseases (transmissible spongiform encephalopathies) are fatal neurodegenerative diseases, including Creutzfeldt-Jakob disease in humans, scrapie in sheep and bovine spongiform encephalopathy in cattle. While genome-wide association studies in human and quantitative trait loci mapping in mice have provided evidence for multiple susceptibility genes, few of these have been confirmed functionally. Phenotyping mouse models is generally the method of choice. However, this is not a feasible option where many novel genes, without pre-existing models, would need to be tested. We have therefore developed and applied an *in-vitro* screen to triage and prioritize candidate modifier genes for more detailed future studies which is faster, far more cost effective and ethical relative to mouse bioassay models. An *in vitro* prion bioassay, the scrapie cell assay, uses a neuroblastoma-derived cell line (PK1) that is susceptible to RML prions and able to propagate prions at high levels. In this study, we have generated stable gene silencing and/or overexpressing PK1-derived cell lines to test whether perturbation of 14 candidate genes affects prion susceptibility. While no consistent differences were determined for seven genes, highly significant changes were detected for *Zbtb38*, *Sorcs1*, *Stmn2*, *Hspa13*, *Fkbp9, Actr10* and *Plg,* suggesting that they play key roles in the fundamental processes of prion propagation or clearance. Many neurodegenerative diseases involve the accumulation of misfolded protein aggregates and ‘prion-like’ seeding and spread has been implicated in their pathogenesis. It is therefore expected that some of these prion-modifier genes may be of wider relevance in neurodegeneration.

## INTRODUCTION

Prion diseases (transmissible spongiform encephalopathies) are fatal neurodegenerative diseases including variant and sporadic Creutzfeldt-Jakob disease (CJD) in human, scrapie in sheep and bovine spongiform encephalopathy in cattle. They are characterized by a long, clinically silent, incubation period and pathologically exhibit neuronal loss, spongiform vacuolation of the neuropil and the accumulation of abnormal forms of the prion protein (often designated PrP^Sc^) in the brain.

Human prion diseases may be sporadic, inherited or acquired, yet genetic susceptibility plays an important role regardless of aetiology. The major genetic component is the prion protein gene (*PRNP*) itself particularly a polymorphism at codon 129 whereby the presence of either methionine or valine, and zygosity, has a profound influence on susceptibility, age of onset and duration of disease (1–3). Despite the primacy of *PRNP*, several studies have now confirmed that many other genes contribute to overall genetic susceptibility. Genome-wide association studies (GWAS) carried out in different human prion diseases including variant CJD (vCJD), iatrogenic CJD, sporadic CJD (sCJD) and kuru identified multiple loci of potential interest (4–6).

Identification of human susceptibility genes in rare diseases is challenging; therefore, parallel studies have also been carried out in mice using incubation time as a phenotype. Mouse models in prion disease are particularly useful as unlike many other neurodegenerative diseases; mice are naturally susceptible to prions and faithfully mirror the clinical and pathological characteristics of the human disease, including the major genetic influence of *Prnp*. Quantitative trait loci (QTL) mapping studies confirmed the influence of genes other than *Prnp* in determining incubation time and additional fine mapping and differential expression studies have suggested numerous candidate genes (7–11).

Prion disease susceptibility genes have been described in many studies in both human and mouse and with varying degrees of statistical confidence; the main challenge is now to verify their role in a functional assay that is relevant to the disease. The standard experimental paradigm is to knockout or overexpress the gene of interest in a mouse model, inoculate with prions and compare all aspects of the phenotype, particularly incubation time, with wild-type controls. Mouse bioassay is costly and time consuming particularly if appropriate mouse models do not already exist. Furthermore, many of these genes are novel with little or no information available in the literature and have no previously known links to prion disease or other neurodegenerative processes. It is not always clear which genes are likely to be the most informative and it is not feasible to actively pursue all these candidates *in vivo* by making new animal models. Where multiple candidate genes require testing, the constraints of mouse bioassays become prohibitive necessitating the development of a fast, reliable, ethical and cost-effective *in-vitro* screen to provide a measure of prion-related function to assist with prioritization for in-depth functional genomics.

Here, we describe an *in-vitro* screening assay that combines the scrapie cell assay (SCA) in prion susceptible neuroblastoma-derived cells (PK1) with silencing or overexpression of candidate genes (12). PK1 cells are a subline of N2a cells highly selected for their susceptibility to infection with the Chandler/RML prion strain and ability to propagate prions at high levels indefinitely. In the standard SCA, RML prions are applied to PK1 cells, the cells become infected and prions are propagated. During this time, cells continue to divide and prions accumulate. Cells are passaged into fresh medium every 3 days at least three times to ensure sufficient dilution that the original inoculum is no longer present. Infectivity is quantified by calculating the number of infected cells using an enzyme-linked immunospot (ELISPOT) assay based on the immunodetection of PrP^Sc^. Proof of principle has already been established that knockdown of *Prnp* in PK1 cells results in the inhibition of prion propagation, suggesting that the application of the SCA combined with manipulation of gene expression may be an effective screening tool (13). In this study, we have generated stable cell lines rather than relying on transient expression of constructs so that knockdown or overexpression is effective throughout the duration of the assay. This allows the detection of gene effects that modify early events such as infectivity uptake and propagation as well as longer term effects such as PrP^Sc^ accumulation, clearance and cell-to-cell spread.

## RESULTS

Candidate genes were selected for functional screening from a variety of sources including human GWAS, mouse differential expression, QTL and association studies (4,11,14–20) (Tables 1 and 2). In the first instance, the aim was to generate at least two stable knockdown cell lines per gene with mRNA expression reduced at least 50% relative to control cells. This was to ensure reproducible results and to minimize the possibility of spurious results due to off-target effects. Green fluorescent protein-knockdown cell lines were generated in parallel as a non-targeting control to ensure that any observed differences were not due to the vector and antibiotic selection. For each gene, at least four shRNA sequences were designed and cloned into the pRetroSuper (Oligoengine) retroviral vector (Supplementary Material, Table S1). Following production of virus, mouse neuroblastoma-derived N2aPK1/2 cells (12) were transduced and grown under puromycin selection to derive stable cell lines. The level of mRNA expression relative to control cells was determined by real-time RT-PCR. Eleven candidate genes were successfully targeted comprising 39 cell lines with a mean mRNA knockdown of 71% (37–98%) relative to the control cell line (Table 1).

**Table 1.** Knockdown cell lines

Gene	Source (M/H)	Cell line	% KD mRNA	SCA fold difference	*P*-value
*Zbtb38*	GWAS (H)	Zbtb38sh1	60	0.24	<0.0001
		Zbtb38sh2	42	0.48	<0.0001
*Sorcs1*	GWAS (H)	Sorcs1sh1	43	0.44	<0.0001
		Sorcs1sh5	37	0.41	<0.0001
		Sorcs1sh6	47	0.27	<0.0001
		Sorcs1sh7	64	0.40	<0.0001
		Sorcs1sh8	54	0.71	<0.0001
*Rchy1*	GWAS (H)	Rchy1sh2	95	–	ns
		Rchy1sh3	88	–	ns
*Stmn2*	GWAS (H) and SNP association (M)	Stmn2sh1	80	0.23	<0.0001
		Stmn2sh4	80	0.21	<0.0001
*Hspa13*	Microarray (M)	Hspa13sh1	70	0.45	<0.0001
		Hspa13sh4	65	–	ns
		Hspa13sh5	63	0.77	0.0007
		Hspa13sh6	53	–	ns
		Hspa13sh7	82	0.52	<0.0001
		Hspa13sh8	85	0.40	<0.0001
*Fkbp9*	Microarray (M)	Fkbp9sh2	96	20.67	<0.0001
		Fkbp9sh3	91	10.23	<0.0001
		Fkbp9sh4	92	9.44	<0.0001
*Actr10*	Microarray (M)	Actr10sh1	61	13.71	<0.0001
		Actr10sh2	84	6.39	<0.0001
		Actr10sh3	66	5.01	<0.0001
		Actr10sh4	71	4.33	<0.0001
*Cbx1*	Microarray (M)	Cbx1sh1	65	–	ns
		Cbx1sh2	65	–	ns
		Cbx1sh3	66	–	ns
		Cbx1sh4	76	–	ns
*Gpr19*	Microarray (M)	Gpr19sh1	92	1.63	<0.0001
		Gpr19sh5	80	0.56	<0.0001
		Gpr19sh6	66	–	ns
		Gpr19sh7	54	–	ns
		Gpr19sh8	61	1.43	0.0007
*Hectd2*	QTL (M) and SNP association (H)	Hectd2sh1	96	2.14	<0.0001
		Hectd2sh3	96	–	ns
		Hectd2sh4	98	1.91	<0.0001
*Sod1*	SNP association (M) and transmission studies	Sod1sh1	67	0.33	<0.0001
		Sod1sh3	77	–	ns
		Sod1sh4	62	–	ns

Bonferonni-corrected significance at 1% is taken as *P*<0.001.

GWAS, genome-wide association study; SNP, single nucleotide polymorphism; QTL, quantitative trait locus; M, mouse; H, human; SCA, scrapie cell assay. SCA mean fold difference and *P*-value are calculated from three independent experiments. SCA fold difference values below 1 indicate a reduction and above 1 an increase in spot number. ns, not significant.

**Table 2.** Overexpressing cell lines

Gene	Source (M/H)	Fold increase mRNA	SCA fold difference	*P*-value
*Rarb*	GWAS (H) and SNP association (M)	nd	–	ns
*Hspa13*	Microarray (M)	140	–	ns
*Fkbp9*	Microarray (M)	9	0.74	<0.0001
*Actr10*	Microarray (M)	4	0.66	<0.0001
*Cbx1*	Microarray (M)	2	–	ns
*Gpr19*	Microarray (M)	5	–	ns
*Hectd2*	QTL (M) and SNP association (H)	13	1.93	<0.0001
*Cpne8*	QTL (M)	nd	–	ns
*Sod1*	SNP association (M) and transmission studies	3	–	ns
*Plg*	PrP binding	nd	2.70	<0.0001

SCA mean fold difference and *P*-value are calculated from three independent experiments. SCA fold difference values below 1 indicate a reduction and above 1 an increase in spot number. Bonferonni-corrected significance at 1% is taken as *P* < 0.001. The level of mRNA expression was similar for all lines reflecting the common CMV promoter. Fold increase in the mRNA expression level was not calculated (nd) where the endogenous levels were very low or undetectable.

GWAS, genome-wide association study; SNP, single nucleotide polymorphism; QTL, quantitative trait locus; M, mouse; H, human; SCA, scrapie cell assay.

For *Rarb*, *Cpne8* and *Plg*, the level of endogenous mRNA expression in N2aPK1/2 cells was either very low or undetectable by real-time RT-PCR rendering them unsuitable for a knockdown approach. cDNA sequences comprising the open reading frames were cloned into the pLNCX2 retroviral vector (Clontech) to generate overexpressing N2aPK1/2 stable cell lines under neomycin selection as above (Supplementary Material, Table S2). Similarly, overexpressing lines were also generated for an additional seven genes to complement the knockdown lines (Table 2). Control cell lines were generated in parallel using the pLNCX2 empty vector. Overexpression was confirmed by real-time RT-PCR. Similar levels of mRNA expression were obtained for all cell lines as directed by the CMV promoter (Table 2).

Each cell line was tested in three independent SCAs with the mouse adapted scrapie prion strain Chandler/RML. The combined results for three independent experiments are shown in Tables 1 and 2 (Supplementary Material, Fig. S1).

*STMN2* was identified as a prion disease susceptibility factor in vCJD, and *ZBTB38* and *SORCS1* were identified in a meta-analysis of vCJD, sCJD and kuru resistance GWAS data (4,14). Highly significant reductions (*P* < 0.0001) in PrP^Sc^ spot number were seen in knockdown cell lines for *Zbtb38* (*n* = 2; x0.24 and x0.48), *Sorcs1* (*n* = 5; x0.44, x0.41, x0.27, x0.40, x0.71) and *Stmn2* (*n* = 2; x0.23, x0.21) (Table 1).

*Hspa13*, *Fkbp9* and *Actr10* were identified in a mouse differential expression study that selected genes based on the correlation between mRNA expression level and incubation time across a range of inbred mice (11). siRNA knockdown of *Hspa13* in chronically infected cells previously resulted in clearance from the cells with ∼40% reduction in PrP^Sc^ positive cells relative to non-targeting control. In this study, six stable knockdown cell lines were generated for *Hspa13* to measure the effect of knockdown on early events such as susceptibility, propagation and cell-to-cell spread rather than just clearance. Four of the six cell lines showed a significant reduction in the number of infected cells relative to the control (0.45-, 0.77-, 0.52- and 0.40-fold difference, Table 1). These data support the previous *in-vitro* clearance data and are consistent with the differential expression data that a decrease in mRNA expression is associated with a longer incubation time (11). An overexpressing cell line was also made for *Hspa13*; however, this did not show any converse increase in spot number.

Three knockdown cell lines for *Fkbp9* were generated with >90% mRNA knockdown and all three showed an increase in the number of PrP^Sc^ positive cells (10–21-fold difference, *P* < 0.0001, Table 1). The *Fkbp9* overexpressing line mirrored these data showing a decrease in PrP^Sc^ spot number (0.74-fold difference, *P* < 0.0001, Table 2). Similarly, four knockdown cell lines for *Actr10* showed an increase in spot number (4–14-fold difference, *P* < 0.0001, Table 1) and this was supported by reduced susceptibility in the *Actr10* overexpressing line (0.66-fold difference, *P* < 0.0001). These data are consistent with the original observation that for both *Fkbp9* and *Actr10*, a lower mRNA expression level correlates with a shorter incubation time (11).

Plasminogen (*Plg*) has been shown to interact with PrP and on addition to both *in-vitro* amplification systems (PMCA—protein misfolding cyclic amplification) and prion-infected N2a cells, has been shown to increase PrP^Sc^ levels (20,21). In contrast, challenge of plasminogen-deficient mice (*Plg^−/−^*) intraperitoneally with RML scrapie prions showed no major effect of plasminogen on survival, although levels of PrP^Sc^ were higher in wild-type mice at the onset of disease symptoms (22). To address the role of plasminogen in prion propagation, we generated a stably overexpressing N2aPK1/2 cell line for inclusion in our screening assay. A 2.7-fold increase in spot number was observed (*P* < 0.0001) thus supporting the previous *in vitro* findings (20).

*Hectd2* was first identified in mouse mapping studies and confirmed as a candidate in human prion disease association studies (15). Three knockdown cell lines, each with mRNA knockdown >95%, were generated. Two of the three lines (Hectd2shRNA1 and 4) showed a significant increase in the number of infected cells (2.14- and 1.91-fold difference, respectively, *P* < 0.0001). Hectd2shRNA3 showed no difference in spot number and it is possible that this may be related to siRNA off-target effects. The *Hectd2* overexpressing line also showed a similar increase in spot number (1.93-fold difference, *P* < 0.0001) which is inconsistent with the data from the knockdown cell lines.

Data for *Gpr19* and *Sod1* knockdowns were inconclusive as significant findings were seen in opposite directions for *Gpr19* and the decrease in spot number for *Sod1* knockdown was seen in only one of three cell lines. These data were not resolved by overexpression as no significant differences were seen for either gene.

No significant differences in spot number were observed either by knockdown or overexpression for *Rchy1*, *Cbx1*, *Rarb* or *Cpne8* despite achieving high levels of knockdown in at least two cell lines (*Rchy1* >88% and *Cbx1* >65%) and consistent levels of overexpression.

The growth rate and differentiation status of N2aPK1/2 cells are factors that may affect the final number of PrP^Sc^ positive cells at the end of the assay. The SCA output is controlled by plating a defined number of cells per well (25 000) onto the ELISPOT plate for final counting; however, growth rates for individual cell lines were also estimated (Supplementary Material, Fig. S2). No differences in doubling times were seen in any of the cell lines tested, suggesting that the differences in PrP^Sc^ spot number observed for *Zbtb38*, *Sorcs1*, *Stmn2*, *Hspa13*, *Fkbp9*, *Actr10* and *Plg* are not artefacts related to growth rate.

PrP expression is central to prion susceptibility and incubation time as demonstrated in mouse models and *in-vitro*, whereby PrP expression is necessary for prion replication and overexpression of PrP in transgenic mice results in a decrease in incubation time (13,23–25). PrP knockdown in PK1 cells significantly reduces susceptibility and cures chronically infected cells. However, cell susceptibility is not increased by increasing the level of PrP expression (11,13,26). Furthermore, comparison of N2a cell clones during selection of N2aPK1 revealed no differences in PrP expression in highly susceptible and resistant clones (12). Based on these precedents, we would not expect the increase in prion-infected cells in some of our lines to be related to an increase in PrP^C^ expression. Nevertheless, it is possible that a decrease in PrP^C^ expression could explain the decrease in spot number. We therefore measured PrP^C^ expression levels by enzyme-linked immunosorbent assay (ELISA) in all cell lines. No differences in PrP^C^ levels were detected in any of our cell lines (Supplementary Material, Fig. S3).

## DISCUSSION

In prion diseases and other conditions including neurodegenerative diseases such as Alzheimer's, genetic modifiers are of considerable interest as a means of identifying risk factors but also to identify rate limiting steps that may provide useful therapeutic targets. For prion diseases, the classical method for evaluating candidate genes is to generate mouse models such as transgenic or knockout animals and to measure aspects of phenotype particularly incubation time following prion inoculation. This will continue to be the method of choice for more detailed pathogenetic studies. However, current genome-wide studies are now generating large numbers of candidate modifier genes that cannot be differentiated further by genetic studies and a higher throughput, more cost-effective and ethical *in-vitro* screen is required to prioritize candidates for in-depth *in vivo* analysis.

The aim of this study was therefore to generate stable cell lines either knocked-down or overexpressing potential modifiers for testing in the SCA as a first round triage to validate candidates and prioritize genes for further studies (12). The candidate gene list for this study was compiled from a diverse range of studies, including linkage to prion disease incubation time in mice, microarray expression and an association with susceptibility to human prion disease. These are complex phenotypes that are likely to be the result of multiple genes, pathways and cellular mechanisms and may also reflect prion strain and route of infection-specific events. It is therefore probable that some genes, which may have a genuine role in prion disease, will not produce a detectable effect in this *in vitro* assay. Only positive results in the SCA are informative, while negatives cannot be interpreted. However, the main objective was to identify modifiers that influence the central processes and key components of prion propagation and cellular clearance, and these are expected to be present in N2aPK1/2 cells as well as expressing the PrP ligands and crucial co-factors required for infectivity and propagation. The cells provide a good model of infectivity uptake, prion propagation, cell-to-cell spread and clearance, although unlike neurons in an *in-vivo* infection they are able to tolerate prion infection without undergoing cell death.

A potential limitation of this assay is that it only reports with RML prions and may not identify strain-specific modifiers. It is therefore possible that strain-specific genes may not report in this assay. This was not considered to be a problem here, as our objective was to identify genes of general importance within the central pathways of prion activity rather than strain-specific modifiers and our aim was to triage genes to prioritize future work thereby accepting that some genuine modifiers may be overlooked. For genes progressed to *in vivo* studies, confirmation of strain-independent effects may be established by bioassay.

In this study, we have tested 14 candidate modifier genes and obtained positive SCA results for seven genes. Given that it is currently unknown whether these candidates are genuine modifiers of prion disease, we are unable to determine the sensitivity, specificity and predictive value of the SCA. False negatives may occur for many reasons including strain-specific factors or interaction with pathways that may not be active in N2aPK1 cells. Similarly, positive results in the SCA may not be replicated in mice due to the inherent differences between a cell and animal model. Alternatively, results may prove to be RML-specific and may not be replicated with other prion strains.

Additional factors that may influence the read out from the SCA are cell viability, differentiation state, growth rate of cells and effects on PrP expression levels. No differences in either growth or PrP expression were observed and experiments were controlled to account for cell numbers and expressed as normalized values to allow for inter-experiment variation.

The identification of susceptibility genes for rare diseases is particularly challenging given the limited number of patients available for study and the threshold for statistical significance in GWAS is very high. In spite of these demands, several human candidate genes have been proposed (4,14). However, the modest effects observed in genetic studies require particularly effective functional tests to differentiate the most promising genes. Five genes derived from human GWAS were tested in our *in-vitro* assay (*ZBTB38, SORCS1, STMN2, RCHY1* and *RARB*), three of which (*ZBTB38, SORCS1* and *STMN2)* showed a highly significant reduction in susceptibility in knocked-down cell lines, providing the first functional evidence of a role in prion disease. This suggests a role in a fundamental aspect of prion disease as these genes were initially identified with human prion strains in case–control studies which may be measuring a range of susceptibility factors including for example uptake of prions from the environment or periphery which are not measurable in this assay. It is possible that the human genes that do not report in this assay have prion strain-specific roles or require allele specific modifications in cells to alter the phenotype. It is also worth noting that the N2aPK1/2 cells are already highly susceptible to prion infection having undergone several rounds of selection. Therefore, as seen with *Prnp* overexpression, it may not always be possible to further increase susceptibility by manipulating certain pathways (12,26). In these cases, negative results are inconclusive.

*ZBTB38* (also known as *CIBZ*) is a zinc finger transcriptional regulator that binds methylated DNA. It has also been identified as a caspase 3 substrate and down regulation of *Zbtb38* in C2C12 cells induces apoptosis (27). RML-infected N2aPK1/2 and stably knocked-down *Zbtb38* cells do not undergo apoptosis, suggesting that an alternative pathway may be affected. *SORCS1* encodes a member of the vacuolar protein sorting family involved in endosomal trafficking and the intracellular sorting of proteins for delivery to the correct cellular location. *SORCS1* has been previously implicated in neurodegeneration by genetic association with Alzheimer's disease as well as with APP processing whereby reduced expression of *SORCS1* is associated with increased γ-secretase processing and Aβ levels possibly as a result of altered trafficking (28). Total PrP^C^ is not affected by *Sorcs1* knockdown in N2aPK1/2 cells; however, it is possible that altered PrP trafficking may reduce its conversion to PrP^Sc^ or facilitate its clearance from the cell. *STMN2* encodes the protein SCGN-10 (neuronal growth associated protein) and is implicated in neurodegeneration through its function related to regulating microtubule stability which is thought to mediate neuronal growth and plasticity (29). While the growth rate of *Stmn2* knockdown N2aPK1/2 does not appear different from control cells, altered microtubule stability may affect cellular trafficking of PrP^C^ and PrP^Sc^.

*Hspa13, Fkbp9, Actr10, Cbx1* and *Gpr19* were identified in a mouse differential expression study and three of these (*Hspa13*, *Fkbp9* and *Actr10*) showed significant differences in PrP^Sc^ spot numbers in the SCA. Hspa13 is a Ca^2+^ induced member of the Hsp70 ATPase protein chaperone family. A significant reduction in infected cells was seen in four of six *Hspa13* knockdown cell lines, supporting the previously described curing of chronically infected cells by siRNA knockdown and transgenic mouse data showing a decrease in incubation time (11). Cell lines Hspa13sh4 and Hspa13sh6 did not show a reduction in spot number which may reflect the less efficient mRNA knockdown in these lines. Hspa13sh5 shows a significant reduction in spot number even though the level of mRNA knockdown is similar to that of Hspa13sh4. However, the number of infected cells is ∼20% higher than for Hspa13sh1, 7 and 8. No corresponding increase in spot number was seen in the Hspa13 overexpressing cell line. Similarly, knockdown of *Prnp* abolishes prion propagation in the SCA, but overexpression does not increase the number of PrP^Sc^ positive cells, thus suggesting that Hspa13 may interact with the same pathway (26).

For both *Fkbp9* and *Actr10* knockdown cell lines, a significant increase in spot number was observed and the converse was seen in their respective overexpressing cell lines. Given that N2aPK1/2 cells are already highly susceptible to RML prions and that increasing *Prnp* expression does not increase cell susceptibility, it was anticipated that not all potential increases in susceptibility would be detectable in the SCA. It is therefore likely that Fkbp9 and Actr10 are increasing susceptibility through an alternative pathway. Fkbp9 is a peptidyl–prolyl isomerase and members of this protein family have been implicated in neurodegeneration, including through accelerating fibrillization (30–32). No change in PrP^C^ levels are seen in Fkbp9 cells; however, it is possible that Fkbp9 isomerase activity may alter the susceptibility of PrP^C^ to conversion. Actr10 (also known as Arp11) forms part of the pointed end complex of the dynactin complex which is the dynein activator complex and is therefore involved in the retrograde transport of cargos along microtubules (33). The participation of three of our candidates (*Sorcs1*, *Stmn2* and *Actr10*) in trafficking identifies a common pathway central to PrP^Sc^ accumulation in cells.

Overexpression of plasminogen (*Plg*) showed an increase in PrP^Sc^ positive cells which is consistent with previous findings that addition of plasminogen to N2a cell cultures increase PrP^Sc^ levels following RML infection (20). These *in vitro* data are in contrast to data from plasminogen-deficient mice that showed no major effect of plasminogen on survival (22). These data highlight the difficulty in assessing the contributions of modifiers and reinforce the need for evaluating their function in a variety of model systems.

In conclusion, we have generated stable knockdown and/or overexpressing cell lines as part of an *in vitro* triage to test the effect of modulating mRNA expression level on susceptibility and prion propagation in the SCA for 14 candidate genes. No consistent differences were seen for seven genes. In spite of the limitations of this assay, it has proved to be a successful triage with changes detected in at least two independent lines for *Zbtb38*, *Sorcs1*, *Stmn2*, *Hspa13*, *Fkbp9, Actr10* and *Plg*. Based on these data, we will pursue mouse models for future work particularly for *Fkbp9*. Since all the major neurodegenerative diseases involve accumulation of misfolded host-encoded proteins and ‘prion-like’ seeded fibrillization may be relevant to their pathogenesis and tissue spread (34–36), it is expected that a proportion of these prion-modifier genes may be of wider relevance in neurodegeneration.

## MATERIALS AND METHODS

### Cloning

19mer shRNA sequences were designed using http://www.thermoscientificbio.com/design-center/, last accessed on 19 May 2014 or http://www.sirnawizard.com/, last accessed on 19 May 2014 design_advanced.php excluding sequences with C at position 1, runs of two or more T at 3′ end or runs of four of the same base and where possible avoiding runs of 3 A/U. shRNA oligonucleotides were designed to be inserted into pSUPER.retro.puro (Oligoengine) at the *Bgl*II and *Hin*dIII sites with a hairpin (5′-TTCAAGAGA-3′) separating the reverse complement 19mer shRNA. Oligos are designed to form a stem loop structure using the following sequences: top strand-5′GATCCCC target sequence (sense) TTCAAGAGA target sequence (antisense) TTTTTA 3′ and bottom strand-5′AGCTTAAAAA target sequence (sense) TCTCTTGAA target sequence (antisense) GGG 3′. Complementary single-stranded oligonucleotides were annealed in annealing buffer [1 mm Tris–HCl (pH 7.5), 0.1 mm EDTA, 5 mm NaCl] by heating to 95°C for 2 min and cooled slowly to room temperature. For overexpression, cDNA for the gene of interest covering the open reading frame was PCR amplified from C57BL/6J mouse brain cDNA library (11) or plasmid (Source Bioscience) and inserted into the multiple cloning site of the retroviral vector pLNCX2 (Clontech).

### Cell culture

N2aPK1/2 cells are highly susceptible to the Chandler/RML strain of prions and were previously subcloned from the N2a cell line (12). N2aPK1/2 cells were cultured in Opti-MEM, 10% FBS (Invitrogen), PenStrep (100 U/ml penicillin, 100 ug/ml streptomycin; Invitrogen). Phoenix Ecotropic, φ-NX Eco, packaging cells (Insight Biotechnology) were cultured in complete Dulbecco's modified Eagle medium (Invitrogen) supplemented with 10% (v/v) Heat Inactivated FBS (Invitrogen) and 1% (v/v) PenStrep (100 U/ml penicillin, 100 ug/ml streptomycin; Invitrogen).

### Generation of stable knockdown and overexpressing cells

Phoenix Ecotropic, φ-NX Eco, packaging cell line (Insight Biotechnology) was used to generate Moloney murine leukaemia virus (MMLV) pseudotyped retroviral supernatants (37) that were then used to infect N2aPK1/2 target cells. 1×10^6^ Phoenix Eco cells were transfected with 7 µg pLNCX2 or pSuperRetro plasmid using Fugene HD (Promega) transfection reagent. Viral supernatant was collected 48 h post-transfection and added to 1 × 10^6^ N2aPK1/2 cells in the presence of 8 µg/ml polybrene (Millipore) for 6 h and placed under drug selection [4 µg/ml puromycin or 500 ng/ml G418 (Sigma)] 48 h post-infection. Transduced cells were cultured in drug selection media for 2 weeks before gene expression analysis for stable knockdown or overexpression then maintained in drug selection media.

### mRNA expression

A total of 10 000 cells were lysed using the Taqman^®^ Gene Expression Cells to CT kit (Ambion, Life Technologies) and RNA reverse transcribed according to the manufacturer's protocol. The resultant cDNA was then assayed by real-time quantitative reverse transcription PCR using Fam-labelled TaqMan Gene Expression assays (Life Technologies) in a duplex reaction using Vic-labelled mouse GAPDH (Life Technologies) as an endogenous control on the Applied Biosystems 7500 Fast Real-Time PCR machine.

### Scrapie cell assay (SCA)

All cell lines were infected with Chandler/RML prions and cultured for 3 weeks, up to five 1:10 splits, before being assayed for prion propagation as described previously (12). Briefly, 18 000 cells of each cell line were seeded in 6–12 wells in a 96-well plate 24 h before exposure to RML homogenate (3 × 10^−5^ to 1 × 10^−7^ dilutions) and control brain homogenate (non-infected CD-1 mouse) for 3 days. Cells were then split 1:10 every 3 or 4 days and assayed after four and five splits by ELISPOT assay. For ELISPOT, 25 000 cells were plated and fixed (50°C for 1 h), lysed and treated with protease K (12). Protease K resistant PrP (PrP^res^) was then detected with ICSM18 monoclonal anti-PrP antibody followed by alkaline phosphatase-linked anti-IgG1 antiserum and visualized with alkaline phosphatase conjugate substrate (Bio-Rad). PrP^res^ positive cells were counted using the Bioreader 5000-Eβ (BioSys).

### Cell growth

To measure the growth of the stable cell lines, cell numbers were counted at two time points 3 or 4 days apart and the cell doubling time calculated using the equation: cell-doubling time (h) = *t*/LOG(*N_t_*/*N*_0_,2), where *N*_t_ = cell number at endpoint, *N*_0_ = starting cell number, *t* = time (h).

### Quantification of PrP^C^

Cells were lysed in 100 mm Tris (pH 7.4), 150 mm NaCl, 1 mm EGTA, 1 mm EDTA, 1% Triton, 0.5% Deoxycholate, 1 mm PMSF, with phosphatase inhibitor and protease inhibitor tablets (Roche) to measure endogenous PrP^C^ levels by ELISA as previously described (38). Following a 1:10 dilution of the lysis buffer, total protein concentration was measured using a Coomassie (Bradford) protein assay kit (Bio-Rad) according to the manufacturer's instructions.

### Statistical analysis

For each cell line, data from three independent SCAs (10–12 wells per assay) were normalized by the spot number obtained for the relevant control cell line for statistical analysis using either a *t*-test or non-parametric Mann–Whitney test. Bonferonni-corrected significance at 1% is taken as *P* < 0.001 (for 14 genes). Statistical tests were carried out using GraphPad InStat (GraphPad Software, Inc, California, USA) and SPSS (IBM).

## SUPPLEMENTARY MATERIAL

Supplementary Material is available at *HMG* online.

## FUNDING

This work was funded by the UK Medical Research Council (MRC). Funding to pay the Open Access publication charges for this article was provided by University College London (UCL).

## Supplementary Material

Supplementary Data
