# Supplementary material for: In vitro screen of prion disease susceptibility genes using the scrapie cell assay
Source: Hum Mol Genet. 2014 May 15;23(19):5102–8. doi: 10.1093/hmg/ddu233 (PMC4159154; doi:10.1093/hmg/ddu233)
Supplement: Supplementary Data [file supp_23_19_5102__index.html]

In vitro screen of prion disease susceptibility genes using the scrapie cell assay — In vitro screen of prion disease susceptibility genes using the scrapie cell assay — Supplementary Data 

# *In vitro* screen of prion disease susceptibility genes using the scrapie cell assay

## Supplementary Data

Supplementary Data

**Files in this Data Supplement:**

- Supplementary Data - Docx file
