# Supplementary material for: In vitro screen of prion disease susceptibility genes using the scrapie cell assay
Source: Hum Mol Genet. 2014 May 15;23(19):5102–8. doi: 10.1093/hmg/ddu233 (PMC4159154; doi:10.1093/hmg/ddu233)

**Supplementary figure legends**

**Figure S1**

Stable gene silenced (shRNA) or overexpressing (LNCX) N2aPK1 cell lines were tested in the SCA (1x10^-5^ RML dilution) together with a control cell line (*GFP* shRNA or LNCX vector). PrP^Sc^ spot numbers from three independent assays are shown normalised to the control line (which precedes it in the graph) ± sem. * P<0.001. A, *Zbtb38*; B, *Sorcs1*; C, *Rchy1*; D, *Stmn2*; E, *Hspa13*; F, *Fkbp9* knockdown; G, *Fkbp9* overexpression; H, *Actr10* knockdown; I, *Actr10* overexpression; J, *Cbx1*; K*, Gpr19*; L, *Hectd2*; M, *Sod1*; N, *Rarb*; O, *Cpne8*; P, *Plg*.

**Figure S2**

Cell growth was measured by calculating the cell doubling time during each SCA. The mean and standard deviation of cell doubling time in hours from at least three independent experiments of knockdown cell lines (A and B) and overexpressing cell lines (C) are shown. There was no significant difference between any of the cell lines and the corresponding control cell line (which precedes it in the graph).

**Figure S3**

PrP^c^ levels in the stable cell lines were quantified by ELISA. The mean and standard deviation are shown for ELISAs performed on three independent cell lysates for each cell line. PrP^c^ levels relative to the appropriate control cells are displayed for the knockdown cell lines (A and B) and overexpressing cell lines (C).

**Table S1 – shRNA sequences for gene silenced cell lines**

| **shRNA name** | **Sequence** |
| --- | --- |
| Zbtb38 shRNA1 | GAGCAAGAAGACGGAGAAA |
| Zbtb38 shRNA2 | AGAAGAAGAAGAAGAGAAT |
| Sorcs1 shRNA1 | TGGATGGAGTTGTGGTCCT |
| Sorcs1 shRNA5 | GAACGGAACCCGAGAAGAT |
| Sorcs1 shRNA6 | GGATCCTGGCATACAGTCA |
| Sorcs1 shRNA7 | AGATTATGTTACTCACAGA |
| Sorcs1 shRNA8 | ACAGCAGCGTGATTCTCAT |
| Rchy1 shRNA2 | AGTCACATTCATCGAAATA |
| Rchy1 shRNA3 | TAAGACAGTCCTTTGAATA |
| Stmn2shRNA1 | TATAATGGATCATGCGATA |
| Stmn2shRNA4 | TGAAGATGGAACAGATTAA |
| Hspa13shRNA1 | AGGAAGTTCTAGACCAATA |
| Hspa13shRNA4 | AGAATGGAGCAGATAAATA |
| Hspa13shRNA5 | GACCAATATAGGCAACTTA |
| Hspa13shRNA6 | GAACAGATTGTGCCTCTTA |
| Hspa13shRNA7 | GAGAGAGAGCCGATGTGAT |
| Hspa13shRNA8 | GGTAGCCATCCGCACTTA |
| Fkbp9shRNA2 | TGAGATAGTTCATGCAAAT |
| Fkbp9shRNA3 | AGTGGAATATAGTTGGAAA |
| Fkbp9shRNA4 | GTACATGTTCATATGGAAT |
| Actr10shRNA1 | AGTGGATGCTTGTGAGATA |
| Actr10shRNA2 | TGGCAGAGAATTTGGTAAT |
| Actr10shRNA3 | TATGCAAGGTGTAGAAATA |
| Actr10shRNA4 | GATCAGGAGTTCAAGGCAA |
| Cbx1shRNA1 | TGAAGATAAAGGAGAGGAA |
| Cbx1shRNA2 | CAGCATATGTTGTCACCAA |
| Cbx1shRNA3 | CTTCATGACTATAGATGGA |
| Cbx1shRNA4 | AGGAATTGGCATTATGTAT |
| Gpr19shRNA1 | GTTATGTGCCAGAGATTAA |
| Gpr19shRNA5 | GGACAGAGCTTCAGTACGA |
| Gpr19shRNA6 | GAGCAAGACTACAAGAAGA |
| Gpr19shRNA7 | GAGCAGGACCATAACCAAA |
| Gpr19shRNA8 | GAGCACAGCTGGATGAGCA |
| Hectd2shRNA1 | AGACAAGTACAGTAAGAAT |
| Hectd2shRNA3 | GATGATTGTACAAGAGTAT |
| Hectd2shRNA4 | AGACCAAACTAGTCATTGA |
| Sod1shRNA1 | AGGATTAACTGAAGGCCAG |
| Sod1shRNA3 | TGAAGAAAGTACAAAGACT |
| Sod1shRNA4 | AAGAGAGGCATGTTGGAGA |

**Table S2. Mouse mRNA transcripts cloned into pLNCX2**

| **Gene** | **Transcript mRNA or clone** |
| --- | --- |
| *Rarb* | NM_011243.1 |
| *Hspa13* | NM_030201.2 |
| *Fkbp9* | NM_012056.1 |
| *Actr10* | NM_007622.3 |
| *Cbx1* | NM_019785.2 |
| *Gpr19* | BC021648.1 |
| *Hectd2* | NM_172637.2 |
| *Cpne8* | NM_025815.2 |
| *Sod1* | NM_011434.1 |
| *Plg* | Image clone 4193957 |

Primers designed to mRNA transcripts for amplification of open reading frame from C57BL/6J brain cDNA or from plasmid clone.


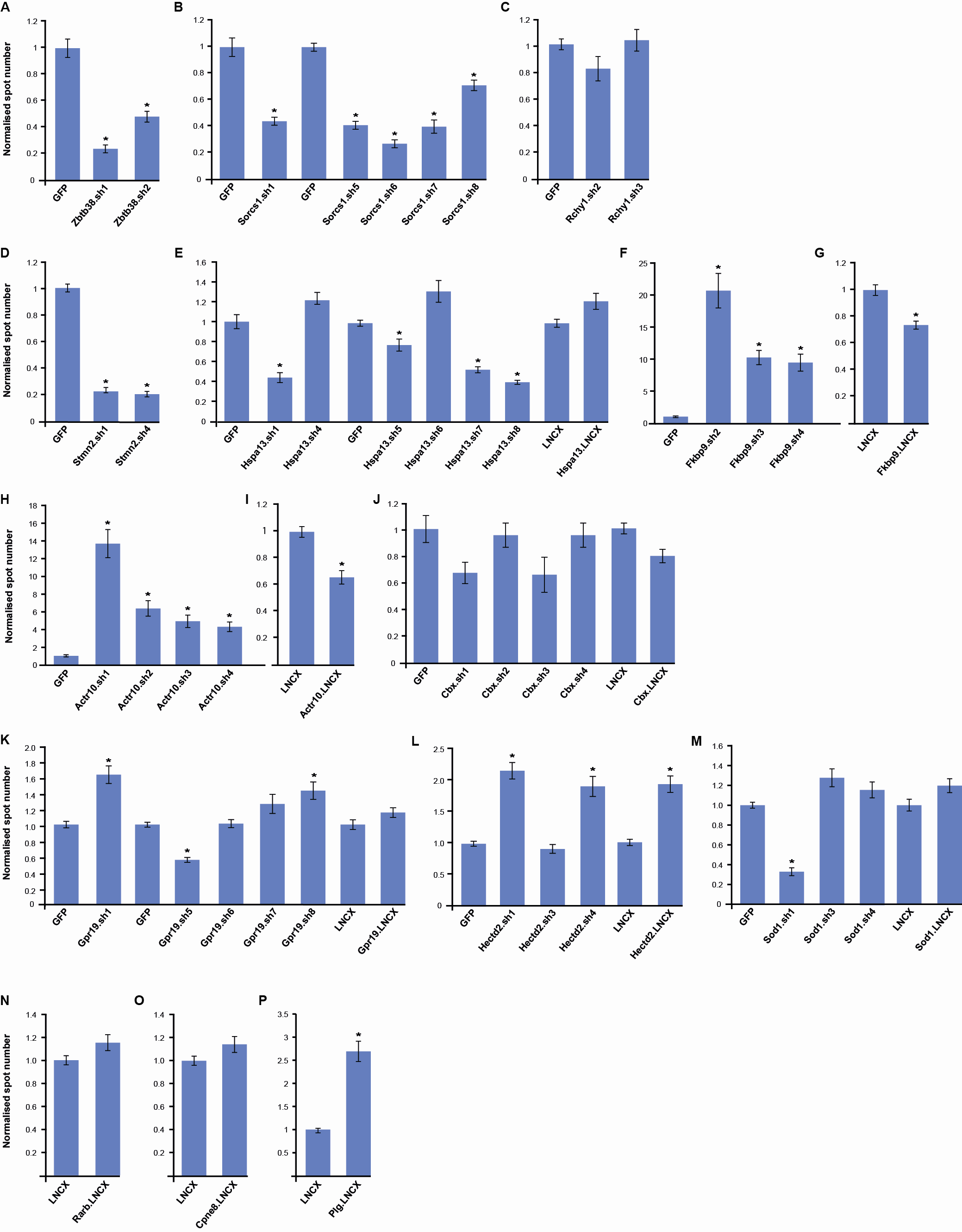

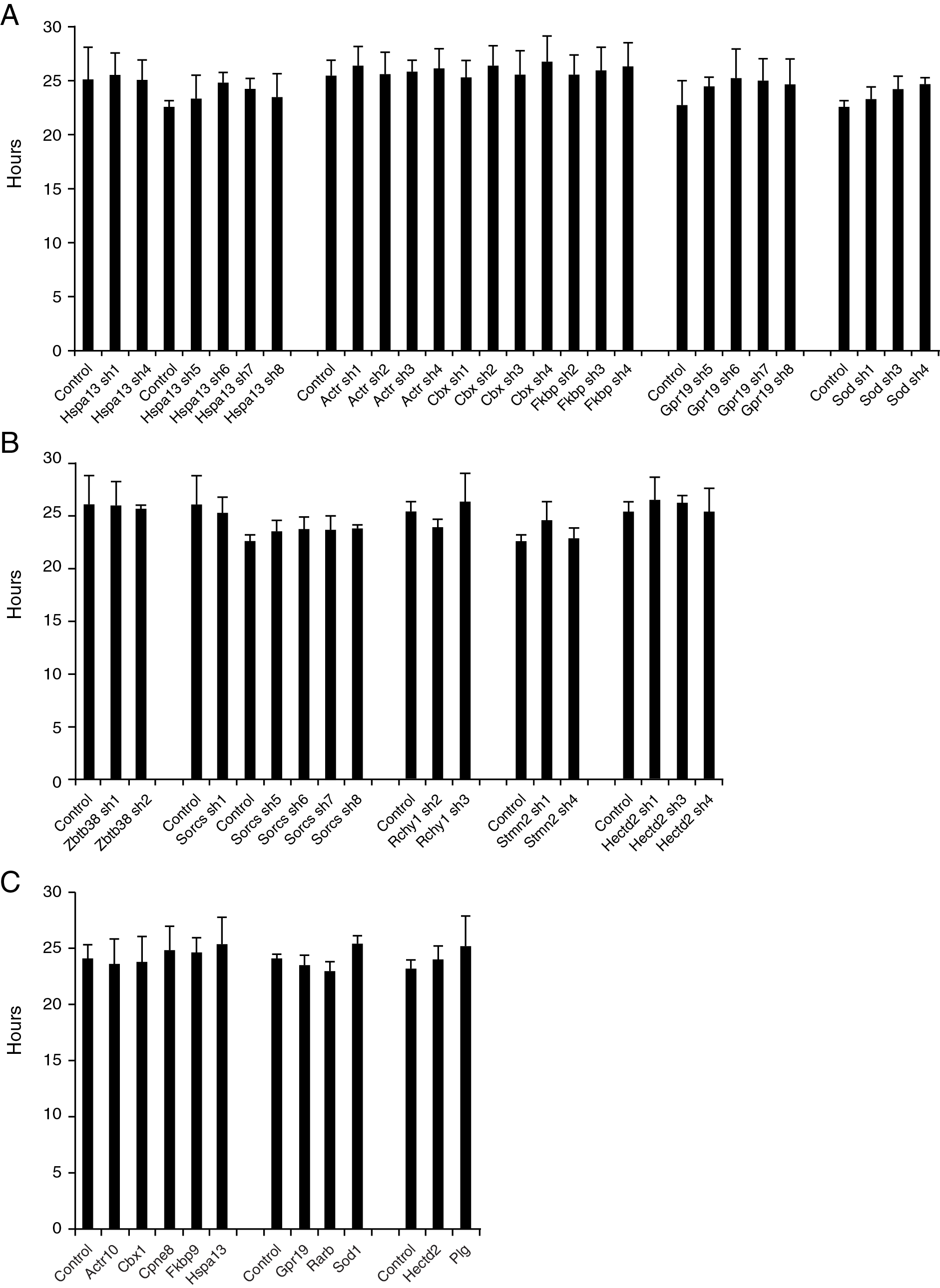

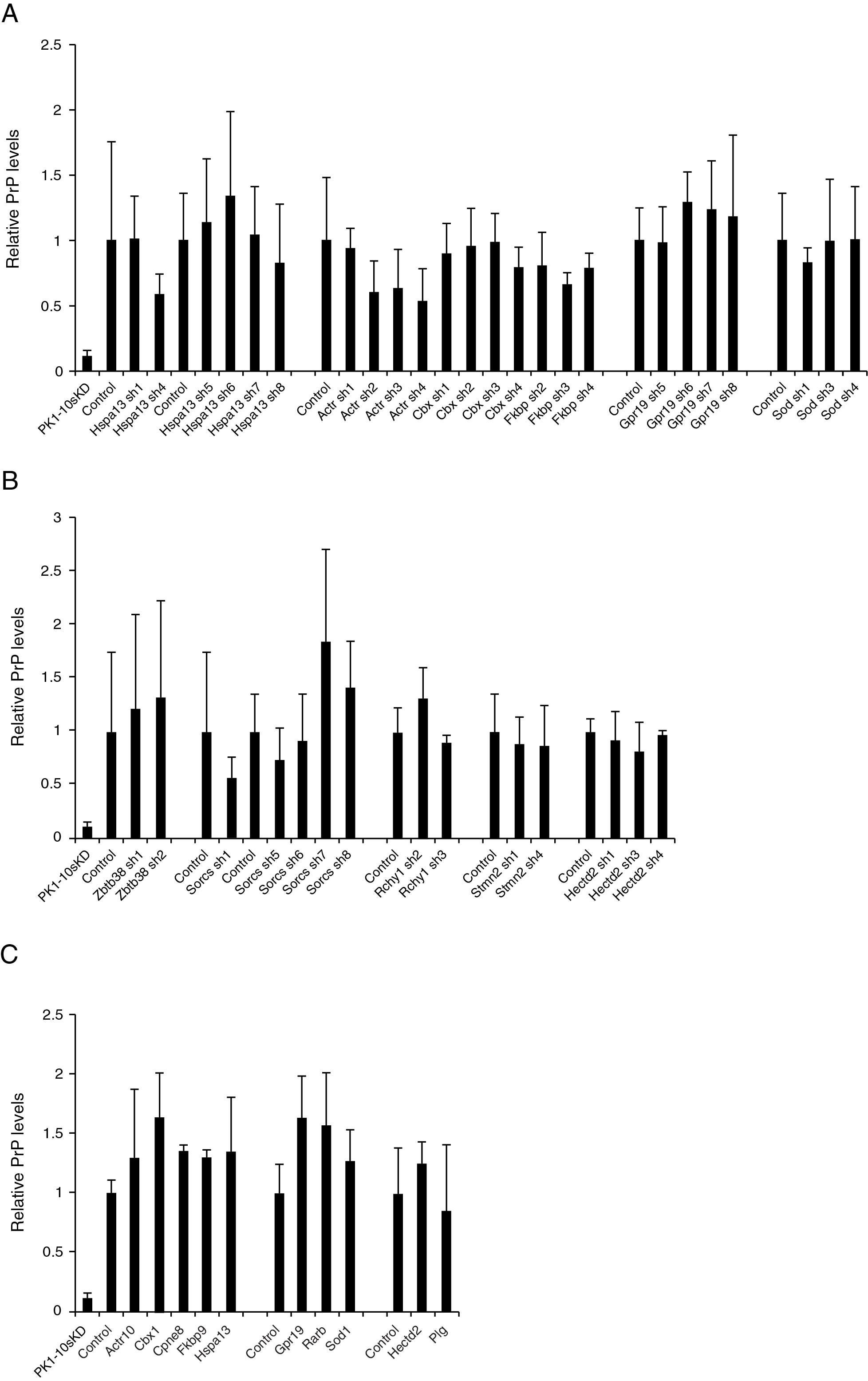

Supplement: Supplementary Data [file supp_ddu233_ddu233supp.docx]
